# Development of a pilot cartilage surgery register

**DOI:** 10.1186/s12891-017-1638-6

**Published:** 2017-06-30

**Authors:** Cathrine Nørstad Engen, Asbjørn Årøen, Lars Engebretsen

**Affiliations:** 1 Oslo Sports Trauma Research Center (OSTRC), Norwegian School of Sports Sciences, postboks 4014 Ullevål Stadion, 0806 Oslo, Norway; 2 Institute of Clinical Medicine, Faculty of Medicine, University of Oslo, Oslo, Norway; 3 Department of Orthopedic Surgery, Akershus University Hospital, 1478 Lørenskog, Norway; 4 Institute of Clinical Medicine, Campus Ahus, University of Oslo, 1478 Lørenskog, Norway; 5 Department of Orthopedics, Oslo University Hospital, Postboks 4956 Nydalen, 0424 Oslo, Norway

**Keywords:** Cartilage surgery, Articular cartilage, Cartilage repair, Knee, Register

## Abstract

**Background:**

Norway has no prospective surveillance system to monitor the outcome of knee cartilage surgery. In 2004 the Norwegian Registry of Knee Ligament (NKLR) was successfully established, and has yielded useful information on the treatment of patients with both knee ligament and combined knee injuries. Patients with focal cartilage defects (FCDs) in their knees have reduced function and the treatment is difficult. There are geographical variations in treatment, and the generalizability from randomized controlled studies is low. These patients would benefit from a standardized long-time follow-up through a cartilage surgery register.

The aim of the present study was to describe the development and report baseline challenges during the setting up of a pilot of a knee cartilage surgery register.

**Methods:**

The study was designed as a prospective cohort study in the form of a register. Patients with full-thickness FCDs in the knee with International Cartilage Repair Society (ICRS) grade 3–4 on arthroscopy were included. The pilot included two hospitals; Oslo University Hospital (OUS), Ullevål and Akershus University Hospital (Ahus).

**Results:**

We registered 58 patients with isolated FCDs, whereas 16 additional patients with full-thickness FCDs were registered through the NKLR. The patient cohort of patients with isolated FCDs consisted of 65% men and had a mean age of 29.8 years. The data are incomplete and the compliance varies from 18 to 73%. The distribution of mean KOOS scores were similar to previous patient cohorts with FCDs, with low scores for the KOOS Sport/Recreation and Quality of Life subscales.

**Conclusion:**

The level of compliance demonstrated a large difference between the two participating hospitals. The compliance for the isolated FCDs were low in both locations, although it reached an acceptable level in one hospital when patients with combined injuries from the NKLR were included. The forms were incompletely filled out by the surgeons postoperatively and need to be revised prior the establishment of a nation-wide register.

**Electronic supplementary material:**

The online version of this article (doi:10.1186/s12891-017-1638-6) contains supplementary material, which is available to authorized users.

## Background

Patients with Focal cartilage defects (FCDs) are young, [1–3] they have increased risk of knee osteoarthritis (OA) [4] and the treatment is challenging. Several questions exist as to which surgical technique, if any, should be offered and whether surgery is better than non-surgical treatment? The lack of evidence within parts of this field suggests that many patients are treated based on surgeons preferences rather than evidence-based medicine.

Randomized controlled trials (RCTs) are a natural part of an evolving clinical research field, but the field of FCDs seems to be demanding as the patient population is heterogeneous, and there are many different surgical techniques. Also, cartilage surgery has still not adequately been compared to non-surgical treatment. The results from RCTs are somewhat inconsistent [5–14] and the RCTs demonstrate low methodological quality [15]. The external validity is also low, [16] and the results from RCTs are thereby not easily applied to a clinical setting.

Orthopedic registers have been successful in Norway and Scandinavia, with high quality and acceptable compliance. [17] The completeness of the NKLR was 97% 21 months after establishment. [18] Compliance is an important part of valid data, and clinical results from the NKLR have already led to changes in treatment [19, 20].

A cartilage surgery register, or rather a prospective cohort study, on a non-biased patient population, will be beneficial for the treatment of these patients. A register will follow trends in surgical treatment and allow feedback on the results to participating hospitals. The quality of treatment will increase through the reporting system. Surgical procedures and devices which result in an unacceptable outcome at an early stage may also be identified. Furthermore, this research design is valuable to identify prognostic factors, whereas an RCT will not be able to determine the influence of several potential important prognostic factors, such as overweight, age, previous surgery, and localization of the defect. Orthopedic registers [21, 22] increase the quality of the treatment in certain patient populations, and we want to explore the potential benefits and challenges in a knee cartilage surgery register. Patients with FCDs of the knee have subtle clinical symptoms, the treatment options are many and varied, and the patient population is heterogeneous, even compared to other orthopedic patient populations. It is therefore necessary to perform a pilot prior to the establishment of a nation-wide cartilage surgery register. In order to explore challenges related to inclusion and logistics and to calculate an expected compliance.

## Methods

### Design and study cohort

The project was designed as a prospective cohort study with follow-up at 5 and 10 years. The inclusion and exclusion criteria are outlined in Table 1. We aimed to include all isolated FCDs. If additional FCDs or degenerative changes were present in other compartments, we still included the patients. If there was a state of generalized knee OA they were excluded as they had reached the end-stage disease. Participation was voluntary, and a written, informed consent was signed before surgery. Two hospitals recruited patients over a 6–8 months period in 2010. The patient pool is thereby restricted to the geographic areas that these hospitals serve, which is approximately 1 million people. Furthermore, a few patients were referred from other geographic areas of Norway. We also included patients with FCDs in combination with a surgical reconstruction of the anterior cruciate ligament (ACL). These patients will be considered separately with regards to outcome, but are important to include in a cartilage surgery register as the cartilage defects may not be adequately followed up in trials or registers focusing on the ACL injury or –reconstruction.

**Table 1:** Illustrates the inclusion and exclusion criteria. In Norway, the retirement age is 67 years and we included patients up to this age

*Inclusion criteria* - Diagnosed focal cartilage defect (ICRS grade 3-4) during arthroscopy or open surgery - Operations/reoperations in patients with a known FCD - Age <67 years *Exclusion criteria* - Generalized knee OA - Other systemic diseases with a known increased risk of knee OA, such as rheumatoid arthritis	

### Data collection

We recorded patient demographics, injury variables, findings during operation and surgical techniques, additional injuries at the time of operation and patient reported outcome measures (PROMs). Non-operative treatment was also registered when FCDs were diagnosed during knee arthroscopy but without further surgical intervention. OUS registered patients from 08.02.10–08.10.10, and Ahus registered from 01.10.10–01.03.11. The data from the NKLR were collected by requesting data from patients treated at the participating hospitals within the data collection period with registered full-thickness FCDs. We received the data as both copies of the NKLR-form, completed by the orthopedic surgeon postoperatively, and in a data file on a CD. KOOS data were not obtained from these patients.

The pilot was paper-based, and the cartilage surgery form (Additional file 1) was constructed with the design of the NKLR-form as a model, but with emphasis on FCDs. The form is on one page with chronologic questions regarding the FCDs. The variables were chosen after discussions with experienced orthopedic surgeons from the participating hospitals in order to include all important aspects of FCD. The form was completed immediately after surgery. Most patients completed the KOOS and the Tegner Activity Scale and questions regarding smoking/tobacco status, BMI, use of NSAIDs and sick-leave (Additional file 2) on their clinical evaluation before knee arthroscopy. However, some patients were included *during* knee arthroscopy due to a newly diagnosed FCD. These patients completed the forms postoperatively based on their experience with the knee prior to operation.

The KOOS score is validated for both cartilage injuries [23] and after cartilage repair, [24] and has acceptable test-retest reliability [24]. It consists of 42 items over five subscales; pain, symptoms, activity of daily living (ADL), sports and recreation and quality of Life (QoL). Each subscale is reported individually with a score ranging from 0 to 100, 100 being the best. Reference values for the general population exist [25]. The Tegner activity score [26] is determined by the most demanding activity the patient is able to perform. The score ranges from 0 to 10, 0 being absent from work due to knee function and 10 being individuals competing on high-level in pivoting sports. The average Tegner score from normative data is 5.7 [27].

One person was responsible for collecting the forms at each hospital. The forms were then checked and plotted into SPSS- by the first author of this paper. Incomplete registration files were returned with a request to fulfill the form. If this was not done after one reminder, the form was registered as “missing”.

### End points

The main outcome was the compliance of the registration, which first and foremost reflect the involvement of the orthopedic surgeons. We included both objective and subjective clinical end points. Total knee replacement (TKR) is one obvious hard endpoint, and another is the diagnosis of severe OA (by arthroscopy, MRI or Kellgren and Lawrence-grading (K&L)). The hard endpoints for the NKLR and the National Prosthesis Registry are revision surgery and TKR. Arøen et al. found that 28% of the patients had previous arthroscopic procedures performed to their knees [3]. Revision surgery is not a suitable hard endpoint for cartilage injuries, as many of them already have had previous knee surgery when scheduled for revision. Revision surgery therefore did not lead to exclusion from the register. The study end points are knee OA and KOOS QoL.

### Validity and reliability

High compliance is necessary to justify any register and was therefore the main outcome of the pilot. Low compliance rates might lead to selection bias, and it may be difficult to predict the direction of the bias; patients might be non-compliant either because they are satisfied with the treatment and feel that they do not need any extra follow-up, or because they are dissatisfied and have sought help elsewhere. Maintaining high compliance and including all patients with FCDs is therefore both a challenge and a critical necessity for a register. We calculated the compliance of the pilot register by going through the operation protocol/local databases in each hospital, which we used as the gold standard. We identified all patients who matched the inclusion criteria based on the surgical description from the operation during the inclusion period and then matched those numbers with the records from the registration. The same was carried out for the data included through the NKLR.

The reliability of the cartilage surgery form is an important issue, where a central aspect is the data describing the lesions. The size of the FCD was calculated by the surgeon using a specific caliper, and the localization was reported corresponding to six predefined areas of the knee joint. Concerning the depth of the lesion, there is an ongoing project with aim of testing the reliability of the ICRS-grading of FCDs (Kjennvold, unpublished). The ICRS score is validated for use after cartilage repair [28].

### Statistics

Power analysis was not performed as the project was not an intervention study. We expected to include approximately 150 patients in two hospitals over a six-month period, however only 74 patients were included over an eight-month period.

Descriptive data included the cartilage surgery form (Additional file 1) and PROMs. This data is presented as means and standard deviations or as medians and interquartile ranges for continuous variables. Frequencies and percentages will be used for summary of categorical variables.

The dataset was also examined for associations and correlations among baseline factors and PROMs with scatter plots and correlation analyses. Roos and Lohmander suggested ten points as a clinical relevant change in score [29]. For the follow-up, we will compute survival plots with KOOS QoL of less than 44 as an end-point. A KOOS QoL less than 44 points has been suggested and tested as a tool for “clinically failure” in patients undergoing ACL reconstruction [30]. This may not be appropriate as a measure for the same clinical outcome in patients with FCD, and must be further explored.

## Results

### Descriptive results

We performed descriptive analyses on the patients with isolated FCDs (Table 2). The patient cohort with combined injuries is previously discussed in articles based on data from the NKLR [31]. 70% of patients with isolated FCDs had a single lesion, whereas 16% had three or more. With regards to the localization of the clinical significant defects, 55% were on the medial femoral condyle (MFC), 16% on the lateral femoral condyle (LFC), 10% on trochlea, 19% on patella, and none on the MTP or LTP. Nearly 14% had a known FCD in the contralateral knee. Only one patient were reported to have no previous surgery to the knee, although this information was missing in 56%. 3.9% operative complications were reported. Nearly 50% of patients received antithrombotic prophylaxis whereas 35% received NSAIDs postoperatively.

**Table 2:** Demonstrates descriptive data of the patient population with isolated FCDs

Variable	Result
Sex	65.5% men
Age in mean (range)	29.8 (10–55)
Size in mean cm^2^ (range)	2.49 (0.04–7.02)
Number of defects in mean (range)	1.57 (1–6)
Normal contralateral knee	81%
Pathogenesis	38% acute injuries, 50% degenerative and 12% unknown
ICRS grade	
3	68%
4	23%
Missing	9%

Diagnostic arthroscopies accounted for 22% of the procedures, 55% were primary cartilage surgery, 10% were revision surgery, 2% were other procedures and 10% were missing classification. Debridement accounted for 29% of the cases, microfracture 9%, harvesting prior to transplantation 3%, transplantation techniques 9% and “other techniques” were chosen in 33% of the cases. In 16% the decision was no to perform any surgery. 38% of patients had an additional surgical procedure performed, in addition to cartilage surgery. 90% did not report on tobacco, regular use of NSAIDs or sick-leave.

There were no gender differences in age, size or depth of lesion or number of lesions. We did not find any correlation between age, size of the lesion or ICRS grade. A weak correlation (r^2^ = .02) between age and number of defects (*p* = .003) seemed to exist.

### Compliance

We included 58 patients with isolated FCDs from the two hospitals, whereas an additional16 were included through the NKLR. Table 3 illustrates the registration of patients with isolated FCDs. At OUS the compliance of isolated FCDs was 60%, whereas it was 73% when we included patients with FCDs in combination with ACL-reconstruction. The corresponding numbers for Ahus were 18% and 22%.

**Table 3:** Illustrates the monthly registration of patients throughout 2010. The column at the right side demonstrates the total number of patients included in the pilot with total number of patients with isolated full-thickness FCDs detected in the operation protocols in parenthesis. OUS = Oslo University Hospital and, Ahus = Akershus University Hospital

	Jan	Feb	Mar	Apr	May	June	July	Aug	Sept	Oct	Nov	Dec	No date	Total (by protocol)
OUS	-	4	9	13	3	9	2	2	7	4	-	1	1	55 (105)
AhUS	-	-	-	-	-	-	-	-	-	2	1	-		3 (17)
Total		4	9	13	3	9	2	2	7	6	1	1	1	58

### Cartilage surgery form

Some of the variables of the form demonstrated many missing values (Table 4).

**Table 4:** Outlines some of the variables from the cartilage surgery form with a high level of missing values

Variable	Missing
Previous surgery	50%
Chronic lesion	6.5%
Date of diagnose	42%
Current injury	36%
Current procedure	10%
Table describing lesion	0–9%
Other procedures	56%

### Patient-reported outcome measures

**Fig. 1:**
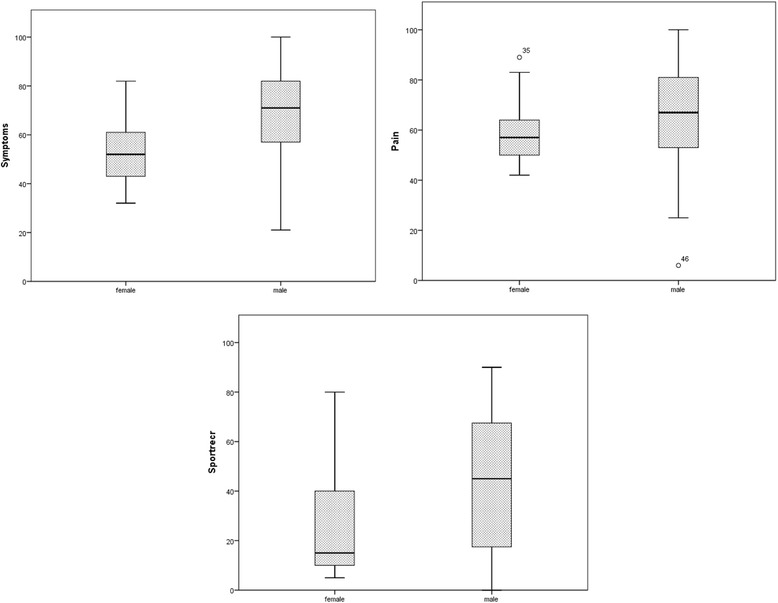
Demonstrates box plots of the results from three subscales of the KOOS with gender as a discriminating variable. The box represents the interquartile range, meaning between the 25th and 75th percentile, while the whiskers represents the range of the data excluding extremes and outliers. The line within the box represents the median. Outliers are marked individually

The mean Tegner score was 4.5 (SD 3.2). The KOOS values are demonstrated in Table 5. We detected gender differences in the symptoms score (p .003 and 95% CI -26.5-(−6.0)) and the sport activity score (p .018 and 95% CI -35.8-(−3.5)) of the KOOS (Fig. 1). Age correlated with Tegner score (p .002), and Tegner correlated with both the sport activity score (p .027) and the QoL score (p .019) of the KOOS. Further, we explored the association between number of defects and the KOOS subscales. The r^2^ was .027 for the subscale “other symptoms”, and the r^2^ was .045 for QoL, indicating that a weak correlation seemed possible. There was, however, no statistical significance.

**Table 5:** Illustrates the results from the KOOS subscales for the patients with isolated FCDs

KOOS value in mean (SD)	
Pain	62.9 (19.7)
Symptoms	62.4 (19.0)
Activity of daily living	73.5 (19.0)
Sports and recreation	37.6 (28.1)
Knee-related quality of life	36.3 (22.6)

## Discussion

This paper describes a pilot for a nation-wide cartilage surgery register. The results show a low compliance and weaknesses in the cartilage surgery form. Worthen et al. suggested ways of ensuring larger patient enrolment and longer follow-up [15]. Other larger cartilage surgery registers have been initiated after this pilot. Similar problems to ours may be encountered also in these registers, however little information exists on this currently.

### Patient population and clinical results

The data describes a patient population with isolated FCDs where 65% were men and with a mean age of 29.8 years. These data are similar to findings from clinical studies on cartilage surgery [32, 33]. The MFC was the most common localization of the FCD, which is also in line with existing clinical studies [34]. There was a weak correlation between age and number of FCDs, which we suspect to be a result from increased degenerative lesions with age. The gender distribution of the patients included from the NKLR was similar with 66% men and a mean age of 34.7 years. The sizes of the lesions are not registered with continuous numbers in the NKLR, but in categories as larger or smaller than 2 cm^2^, and 83.3% were larger than 2 cm^2^. It is therefore likely that they are comparable to the patients with isolated FCDs, but this is not definite. The localization of the defects included from the NKLR differed, as only 33.3% were located on the MFC and 22% were located on the tibial plateau. Nearly 30% had defects on “large parts of the joint”, and this might represent more degenerative changes than what was found in the patients with isolated FCDs.

### Compliance

The compliance was variable. One of the hospitals had 73% compliance for the combination of both patient cohorts. The second hospital registered few patients, but they also had a low total incidence of full-thickness FCDs demonstrated in their surgical protocols over the inclusion period.

The NKLR has a reporting system where hospitals are provided with continuous feedback from the register in an effort to achieve high compliance. The completeness of the NKLR was 97% 21 months after establishment [18]. The 2 year results was decreasing, with lower rates for smaller hospitals [35]. Still, the compliance is nearly 100% at some hospitals, whereas it is 10–20% in others [36]. The reason is not clear, although low motivation among orthopedic surgeons is a possible explanation. NKLR is currently shifting towards electronic registration, and hopefully this will lead to a rise in compliance. The Danish Knee Ligament Reconstruction-registry had a compliance of 60% in 2005 and 86% in 2011 [37]. The compliance for both the joint prosthesis registry and for the femoral neck fracture registry is high. Generally, high-volume hospitals perform better and the same trend is expected for the registration of patients with FCDs. The yearly report of the NKLR (2010) found 60% patient compliance for the KOOS at the 2 year follow-up, suggesting that effort must be made also to raise the patient-response-rate.

In order to increase the compliance of the cartilage surgery register, the fill-out form and the overall logistics are kept simple. The departments and the orthopedic surgeons are regularly reminded to include patients. Increasing the patient-response-rate is done with similar techniques as for other orthopedic registries. Patients are informed of the importance and benefits of a cartilage surgery register. They are assured that their contribution is important, and in this, attempt to provide some ownership to the register as well. We have currently also involved two individuals from the patient organisation. They represent the interests of the patients, and meet regularly to seminars where they may influence on all aspects of the register.

### Cartilage surgery form

We identified several variables with 50% missing values. This may have been due to an unclear way of presenting the variables or a difficult order of the variables. The cartilage surgery form was re-evaluated after the experiences from this pilot study and the updated edition is currently being tested in a second pilot.

### Cartilage surgery register

The current pilot was run in 2010. There are certain individual cartilage surgery registers initiated by the industry and by individual orthopedic surgeons internationally [38]. Genzyme Tissue Repair initiated an international registry in order to assess the effectiveness of ACI. Industrial registers tend to include more advanced cartilage surgery than what is common trends in general [39]. Data from a more recently established register, the German Cartilage Registry, is now available [40, 41]. The ICRS has also recently initiated a cartilage surgery register. Both of the latter registers are established outside of Norway, which restricts our contribution due to restrictions in export of person-identifiable data. Also, none of the existing cartilage surgery registers includes patients undergoing non-operative treatment and less invasive cartilage surgery, such as debridement. There are reasons to believe that patients from the existing registers differ from the general patient population with FCDs of the knee.

In a register where the inclusion criteria is an FCD, we are able to register only FCDs and exclude patients with knee OA. As opposed to the existing electronic registers that rely on registration from ICD-10 codes, the registration is then more robust against over-registration. Duplicates are easily noticed based on personal data and operation date. The data from a register is easily and quickly accessible. The database contains a much bigger pool of patients with increasing opportunities of detecting poor outcomes, correlations and prognostic factors that are not possible to find in strictly controlled studies.

As a cartilage surgery register will include patients with different levels of cartilage surgery, it is possible to find prognostic factors for all levels of treatment. A register will secure long-term follow-up data on this patient population. It is necessary to ensure that these patients are followed for a longer period, as they are still young and have several potential years with both work and recreation/sports. As for ACI it takes 2 years simply for the new tissue to mature [42]. The register will identify failures earlier than what is possible today.

The efficacy of an intervention or a product is studied in an RCT, but its effectiveness can never be assessed in a controlled clinical study [43]. A register makes it possible to find the effectiveness of specified knee cartilage surgery compared to no surgery (or simple debriding techniques). The patients treated non-operatively will not act as true controls as the indication for surgery is probably biased. Nevertheless, comparisons can be made if there is good control of prognostic factors and confounders. High quality prospective cohort studies may complement research gaps. The limitations of both RCTs and the limitations of retrospective analyses make it important to establish a register. However, it is important to include all patients and all level of treatment to avoid selection bias. Registers can also be used in RCTs as described in the field of clinical effectiveness research. Given such an application, a cartilage surgery register will be particular helpful in questions that may otherwise only be answered through costly and challenging RCTs.

### Strength and weaknesses

A weakness of all registries is the internal validity, which will never be as high as a high-quality RCT. However, it is possible to obtain high quality data with a proper design, even in the absence of randomization and blinding. A register is the only method that measures the effectiveness of treatment in the general population. Internal validity is kept high with good control of all other variables.

The challenges concerning suboptimal IT-solutions is another weakness of most quality registries. Data must often be manually written a second time and then transferred to the register. However, electronic solutions are now required for quality registers in Norway. Solutions where the data collection with relevant and predefined data are automatically extracted from the electronic journal system are being developed.

The register is publicly funded. This is important to prevent bias due to commercial interests. There is a strong association between private industrial funding and lower level of evidence where the level of evidence is higher in non-industrial studies [44]. Another challenge is that research becomes more dependent on funding from the industry. In a review by Harris et al., [45] 26% of the studies reported a financial conflict of interest while 40% failed to report the existence of this. The risk of bias decreases with a non-industrial register.

### Future organization

Successful orthopedic registries have been established in several countries for joint replacement surgery (such as Norway, 1987, Sweden, 1975, Finland, 1980, Denmark, 1995, Australia, 1998) and knee ligament surgery (Norway, 2004, Sweden, 2005 and Denmark, 2005). An important issue to discuss is which and how many hospitals to include in the register. Whether the register should be national and include all hospitals, or only hospitals performing advanced cartilage surgery must be addressed. Another solution is to include the largest hospitals within each of the four health regions in Norway or to develop a Scandinavian register. The NKLR cooperates with the Swedish and Danish registries. A cooperation with the NKLR will also be discussed for a potential future cartilage surgery register in Norway.

## Conclusion

We have demonstrated that it is possible to achieve a similar compliance for a cartilage surgery register in one of our participating hospitals, as demonstrated in other successful orthopedic registers. Although, it requires both surgeon participation and awareness of logistical challenges. We are currently running a second pilot in 5 hospitals in Norway, with the revised cartilage form and with a longer registration period, taking into account the lessons learned from this pilot.

## Additional files

Additional file 1: Cartilage surgery report. (PDF 180 kb)

Additional file 2: Patient questionnaire. (DOCX 91 kb)

## Abbreviations

ACL: Anterior cruciate ligament
BMI: Body mass index
FCD: Focal cartilage defect
ICD-10: 10th revision of the International statistical classification of diseases and related health problems
ICRS: International Cartilage Repair Society
K&L: Kellgren & Lawrence system for classification of osteoarthritis
KOOS: Knee injury and osteoarthritis outcome score
LFC: Lateral femoral condyle
LTP: Lateral tibial plateau
MFC: Medial femoral condyle
MRI: Magnetic resonance imaging
MTP: Medial tibial plateau
NKLR: Norwegian Registry of Knee Ligament
NSAIDs: Nonsteroidal anti-inflammatory drugs
OA: Osteoarthritis
PROMs: Patient reported outcome measures
RCT: Randomized controlled trial
TKR: Total knee replacement
