# Supplementary material for: Development of a pilot cartilage surgery register
Source: BMC Musculoskelet Disord. 2017 Jun 30;18:282. doi: 10.1186/s12891-017-1638-6 (PMC5493883; doi:10.1186/s12891-017-1638-6)
Supplement: Supplementary file 1 — Cartilage surgery report. (PDF 180 kb) [file 12891_2017_1638_MOESM1_ESM.pdf]

PILOTSTUDIE BRUSKREGISTER
Forskningsenheten, Ortopedisk Senter
OUS, Ullevål
Kirkeveien 166, Bygg 73, 2. etg
0473 OSLO
Tlf: 992 33 611

F.nr. (11 sifre)..
Navn...
Sykehus..
(Skriv tydelig ev. pasient klistrelapp – **spesifiser sykehus**.)

FOKALE BRUSKSKADER I KNE - diagnoistisert ved artroskopi

BRUSKKIRURGI OG ALLE REOPERASJONER på pasienter som tidligere har skadet/operert brusk i kneet.

Alle klistrelapper (med unntak av pasientklistrelapp) settes i merket felt på baksiden av skjemaet.

(Bilateral operasjon = 2 skjema)
AKTUELL SIDE (ett kryss) ☐⁰ Høyre ☐¹ Venstre

MOTSATT KNE ☐⁰ Normalt ☐¹ Kjent bruskskade

TIDLIGERE OPERASJON I SAMME KNE (ev. flere kryss)
☐ ACL ☐ MCL ☐ PLC ☐ Medial menisk
☐ PCL ☐ LCL ☐ Brusk ☐ Lateral menisk
☐ Annet, spesifiser

AKUTT SKADE
☐ Ja ☐ Nei ☐ Usikkert
SKADEDATO FOR AKTUELL SKADE (mm.åå) |__|__| |__|__|

KRONISK SKADE
☐ Ja ☐ Nei ☐ Usikkert
SYMPTOMVARIGHET (i mnd) ....

DIAGNOSEDATO FOR AKTUELL SKADE (mm.åå) |__|__| |__|__|

AKTIVITET SOM FØRTE TIL AKTUELL SKADE
☐⁰ Fotball ☐⁵ Ukjent
☐¹ Mosjon/friluftsliv ☐⁶ Ikke spurt
☐² Arbeid ☐⁷ Slalåm
☐³ Trafikk ☐⁸ Håndball
☐⁴ Annet ....

AKTUELL SKADE (Registrer alle skader – også de som ikke opereres)
☐ ACL ☐ MCL ☐ PLC ☐ Menisk med.
☐ PCL ☐ LCL ☐ ☐ Menisk lat.
☐ Annet..

OPERASJONSDATO (dd.mm.åå) |__|__| |__|__| |__|__|

AKTUELLE OPERASJON (ett kryss)
(Hvis ingen kryss, gå direkte til ANDRE PROSEDYRER.)
☐ Diagnostisk Artroskopi ☐ Primær bruskbevarende kirurgi
☐ Bruskrevisjonskirurgi

	Omfang					
	Areal (cm²) ≤2	>2	Areal (cm²)	ICRS Grade* (1-4)	Sannsynlig årsak** (1-5)	Behandlingskode*** (1-9)
Patella MF	☐	☐				
Patella LF	☐	☐				
Trochlea fem.	☐	☐				
Med. fem. cond.	☐	☐				
Med. tib. plat.	☐	☐				
Lat. fem. cond.	☐	☐				
Lat. tib. plat.	☐	☐				

***ICRS Grade:** 1 Nearly normal: Superficial lesions, soft indentation and/or superficial fissures and cracks; 2 Abnormal: Lesions extending down to <50% of cartilage depth; 3 Severely abnormal: Cartilage defects extending down >50% of cartilage depth as well as down to calcified layer; 4 Severely abnormal: Osteochondral injuries, lesions extending just through the subchondral boneplate or deeper defects down into trabecular bone.

****Sannsynlige årsaker:** 1 Traume; 2 CM: chondromalacia patellae; 3 OCD: osteochondritis dissecans; 4 OA: primær artrose; 5 Annet: Spesifiser årsak i aktuelle rubrikk
*****Behandlingskoder**: 1 Debridement; 2 Mikrofraktur; 3 Mosaikk; 4 Biopsi til dyrking; 5 Celletransplantasjon; 6 Celletransplantasjon med matrix; 7 Periosttransplantasjon; 8 Ingen behandling; 9 Annet: Spesifiser behandling i aktuelle rubrikk

BRUSKIMPLANTAT
☐ MACI ☐ Chondrogide
☐ Cartipatch ☐ HYAF
☐ Fixasjon OCD ☐ TrueFit
☐ Annet ………………..

ANDRE PROSEDYRER (ev. flere kryss)
☐ Meniskoperasjon ☐ Osteosyntese
☐ Synovektomi ☐ Biopsi
☐ Fjerning av implantat ☐ Operasjon pga infeksjon
☐ Mobilisering i narkose ☐ Bentransplantasjon
☐ Osteotomi ☐ Artrodese
☐ Protese
☐ Annet …………………………………………………..

AKTUELL BEHANDLING AV MENISKLESJON
Hvis meniskreseksjon; angi andel som er fjernet og hvilken menisk........... ☐ Mediale ☐ Laterale ☐ Begge

	Reseksjon	Sutur	Syntetisk fiksasjon*	Menisk-transpl.	Trepanering	Ingen
Medial	☐	☐	☐	☐	☐	☐
Lateral	☐	☐	☐	☐	☐	☐

* Sett klistrelapp på merket felt på baksiden

DAGKIRURGISK OPERASJON ☐ ⁰ Nei ☐ ¹ Ja

PEROPERATIVE KOMPLIKASJONER ☐ ⁰ Nei ☐ ¹ Ja,
hvilke(n) ....

OPERASJONSTID (hud til hud).......................min.

SYSTEMISK ANTIBIOTIKAPROFYLAKSE
☐⁰ Nei ☐¹ Ja, Hvilken (A)...
Dose (A)................Totalt antall doser...............Varighet ....timer
Ev. i kombinasjon med (B)..
Dose (B)................Totalt antall doser...............Varighet ....timer

TROMBOSEPROFYLAKSE
☐⁰ Nei ☐¹ Ja, hvilken type…………………………………………………….

Dosering opr.dag……………………….Første dose gitt preopr ☐⁰ Nei ☐¹ Ja

Senere dosering…………………………………..Antatt varighet……….døgn

Ev. i kombinasjon med ……………………..…………………..……………

Dosering...…………………………………..…Antatt varighet...……….døgn

Annet, spesifiser ……………………………………………………………

NSAIDs
☐⁰ Nei ☐¹ Ja, hvilken type…………………………………………………….

Eventuellle kommentarer

…………………………………………………………………………
…………………………………………………………………………

Lege:...
Legen som har fylt ut skjemaet (navnet registreres ikke i databasen).
