# Supplementary material for: Development of a pilot cartilage surgery register
Source: BMC Musculoskelet Disord. 2017 Jun 30;18:282. doi: 10.1186/s12891-017-1638-6 (PMC5493883; doi:10.1186/s12891-017-1638-6)
Supplement: Supplementary file 2 — Patient questionnaire. (DOCX 91 kb) [file 12891_2017_1638_MOESM2_ESM.docx]

**AKTUELL SIDE** (ett kryss) □^0^ Høyre □^1^ Venstre

**VEKT: ……………**

**HØYDE: ……………**

**RØYKESTATUS**

□ røyker

□ ikke-røyker

**HVIS RØYKER, antall/uke: ………………….**

**BRUKER DU NSAIDs REGELMESSIG MOT SMERTE?** □ Ja □ Nei

**HVIS JA, angi gram/uke: ………………….**

**HAR DU VÆRT SYKEMELDT IFM BRUSKSKADEN I KNEET?**  □ Ja □ Nei

**HVIS JA; hvor lenge? ………………………………**

**HAR DU BLITT OMSKOLERT IFM BRUSKSKADEN I KNEET?** □ Ja □ Nei
